# Tensile Behavior of 3D Printed Polylactic Acid (PLA) Based Composites Reinforced with Natural Fiber

**DOI:** 10.3390/polym14193976

**Published:** 2022-09-23

**Authors:** Eliana M. Agaliotis, Baltazar D. Ake-Concha, Alejandro May-Pat, Juan P. Morales-Arias, Celina Bernal, Alex Valadez-Gonzalez, Pedro J. Herrera-Franco, Gwénaëlle Proust, J. Francisco Koh-Dzul, Jose G. Carrillo, Emmanuel A. Flores-Johnson

**Affiliations:** 1 Facultad de Ingeniería, Universidad de Buenos Aires, Av. Las Heras 2214, Buenos Aires C1127AAR, Argentina; 2 CONICET-Universidad de Buenos Aires, Instituto de Tecnología en Polímeros y Nanotecnología (ITPN), Av. Las Heras 2214, Buenos Aires C1127AAR, Argentina; 3 Unidad de Materiales, Centro de Investigación Científica de Yucatán, Calle 43 No. 130 Col. Chuburná de Hidalgo, Mérida 97205, Yucatán, Mexico; 4 Facultad de Ingeniería, Universidad ECCI, Bogotá 111321, Localidad de Teusaquillo, Colombia; 5 School of Civil Engineering, The University of Sydney, Sydney, NSW 2006, Australia; 6 Sydney Manufacturing Hub, The University of Sydney, Sydney, NSW 2006, Australia; 7 Australian Nuclear Science and Technology Organisation (ANSTO), Lucas Heights, NSW 2234, Australia; 8 School of Mechanical and Manufacturing Engineering, University of New South Wales (UNSW Sydney), Sydney, NSW 2052, Australia

**Keywords:** polylactic acid (PLA), natural fiber, henequen fiber, natural fiber reinforced composite (NFRC), additive manufacturing, 3D printing, mechanical property

## Abstract

Natural fiber-reinforced composite (NFRC) filaments for 3D printing were fabricated using polylactic acid (PLA) reinforced with 1–5 wt% henequen flour comprising particles with sizes between 90–250 μm. The flour was obtained from natural henequen fibers. NFRCs and pristine PLA specimens were printed with a 0° raster angle for tension tests. The results showed that the NFRCs’ measured density, porosity, and degree of crystallinity increased with flour content. The tensile tests showed that the NFRC Young’s modulus was lower than that of the printed pristine PLA. For 1 wt% flour content, the NFRCs’ maximum stress and strain to failure were higher than those of the printed PLA, which was attributed to the henequen fibers acting as reinforcement and delaying crack growth. However, for 2 wt% and higher flour contents, the NFRCs’ maximum stress was lower than that of the printed PLA. Microscopic characterization after testing showed an increase in voids and defects, with the increase in flour content attributed to particle agglomeration. For 1 wt% flour content, the NFRCs were also printed with raster angles of ±45° and 90° for comparison; the highest tensile properties were obtained with a 0° raster angle. Finally, adding 3 wt% content of maleic anhydride to the NFRC with 1 wt% flour content slightly increased the maximum stress. The results presented herein warrant further research to fully understand the mechanical properties of printed NFRCs made of PLA reinforced with natural henequen fibers.

## 1. Introduction

Additive manufacturing (AM), also known as 3D printing, has been increasingly used in the last decade due to its versatility in producing numerous products with complex shapes and specific mechanical properties at a low cost. The increase in the use of 3D printing over the last few years has been driven by the rise in 3D printers’ affordability and the availability of printing materials [1]. Three-dimensional printing has been employed by biomedical, civil engineering, aerospace, and automotive industries to make prototypes, models, spare parts, dental crowns, artificial limbs, etc. [2,3,4]. One of the most popular 3D printing techniques is fused deposition modelling (FDM), which is based on the thermal extrusion process of a thermoplastic filament, which is melted and deposited layer by layer [5]. Subsequently, the deposited material is cooled and solidified, and the bond between the extruded filaments is consolidated. FDM is becoming increasingly popular due to its low cost, low maintenance, and the increasing variety of raw materials for 3D printing, for instance polylactic acid (PLA), polypropylene (PP), polyethylene terephthalate glycol (PETG), and acrylonitrile butadiene styrene (ABS) [6,7]. PLA is a linear aliphatic thermoplastic polyester derived from lactic acid, obtained from renewable resources such as corn or sugarcane fermentation [8,9,10]. PLA is biodegradable, environmentally friendly, and emerging as a substitute for oil-based polymers. Furthermore, green composites have been fabricated using PLA with natural fillers [11,12] or fibers [10,13,14,15]. One of the main benefits of using natural particles and lignocellulosic materials in PLA-based 3D printed materials is their availability, low cost, low weight, and environmental impact reduction.

Composites from renewable resources based on FDM technology have become attractive products for construction [12], automobiles [16], furniture [17], and other consumer applications due to increased environmental awareness and demand for eco-friendly materials [18,19]. Natural-fiber-reinforced composite (NFRC) and wood–plastic composite (WPC) are composite materials consisting of a thermoplastic polymer matrix with a small content of natural fibers [20] and wood [21], respectively. While the mechanical properties of NFRCs and WPCs fabricated by traditional methods such as extrusion or injection molding [20,22] have been extensively studied, the mechanical behavior of 3D printed PLA-based NFRCs and WPCs is still being investigated [5,20,23]. Figueroa-Velarde et al. [24] investigated the tensile and flexural properties of 3D printed PLA reinforced with 3–10% agave fibers, which had a diameter of 37.7 µm and a length of 255 µm. They observed that both tensile modulus and strength decreased with the increase in fiber content compared to pure PLA. They also found a slight increase in the flexural modulus when a fiber content of 3% was used compared to pure PLA. Matsuzaki et al. [25] studied the tensile properties of 3D printed PLA reinforced with continuous jute fibers. They found that the tensile modulus, tensile strength, and strain to failure of the 3D printed reinforced composites increased compared to those of pure PLA. Hinchcliffe et al. [26] investigated 3D printed PLA-based ducted I-beams, in which continuous flax fiber strands with 0.5 mm diameter were threaded through the ducts. They observed that pre-stressing the fibers resulted in an improvement in flexural properties compared to unreinforced specimens. Depuydt et al. [27] studied the tensile properties of filaments for 3D printing made of PLA compounded with plasticizer and bamboo fibers. They observed that the tensile modulus of the reinforced filaments increased by 215% when long bamboo fibers with a median diameter of 254 µm and length of 2015 µm were used. However, no strength increase was observed in the reinforced filaments. Liu et al. [28] studied the tensile strength of 3D printed PLA composites with pine wood flour with particle sizes of <50 μm. They found that the tensile strength increased with an increased wood flour content of up to 15% and a decrease for wood flour contents higher than 15%. Baghia et al. [29] investigated the tensile properties of 3D printed wood-filled PLA samples containing 20% ball-milled wood flour from poplar trees; the flour particles had a median diameter of 43 μm. They found that the tensile modulus for the 3D printed reinforced samples increased compared to pure PLA, while the tensile strength and strain at break decreased. Kariz et al. [30,31] studied the tensile strength of filaments for 3D printing made of PLA with 10–50% beech wood content with particle sizes of <237 μm. They reported that the tensile strength of the filaments increased with a 10% content of wood; however, a higher content of wood resulted in a decrease in the tensile strength. Tao et al. [32] investigated the tensile behavior of 3D printed PLA/wood flour specimens with a flour content of 5% with a mean particle size of 14 μm. They found that the tensile stress increased for strains lower than 1.5% and decreased for larger strains compared to pure PLA.

Based on the literature mentioned above, studies addressing the tensile properties of 3D printed PLA-based composites reinforced with natural fibers are scarce. Moreover, research on using henequen fibers (*Agave fourcroydes* Lem.) to reinforce 3D printed NFRC has not previously been reported to the authors’ knowledge. The henequen plant is endemic to the Yucatan peninsula in Mexico. Henequen fibers are lignocellulose fibers with a high cellulose content (60%) extracted from the leaves of the henequen plant. These fibers are used to fabricate ropes, mats, sacks, and bags [33]. In addition, henequen fibers have been used to reinforce polymer composites [34,35] and cement-based composites [36,37]. Furthermore, some studies have reported the mechanical and thermal properties of PLA/sisal fiber composites [38,39], where the sisal plant is a close relative of the henequen plant [40]. This work characterizes the tensile behavior of 3D printed NFRCs reinforced with different contents of henequen fiber flour. In addition, differential scanning calorimetry (DSC) analysis and microscopic characterization of the printed PLA and NFRCs are also presented.

## 2. Materials and Methods

### 2.1. Materials

#### 2.1.1. Polylactic Acid (PLA)

Extrusion-grade PLA Ingeo 2003D in pellet form (NatureWorks, USA) derived from renewable resources was used in this work. According to the manufacturer, the PLA material has a density of 1240 kg/m^3^, a melt flow index of 6 g/10 min (210 °C, 2.16 kg), a melting temperature of 210 °C, a tensile elastic modulus of 3.5 GPa, and a tensile strength of 60 MPa [41].

#### 2.1.2. Henequen Fibers

**Figure 1:**
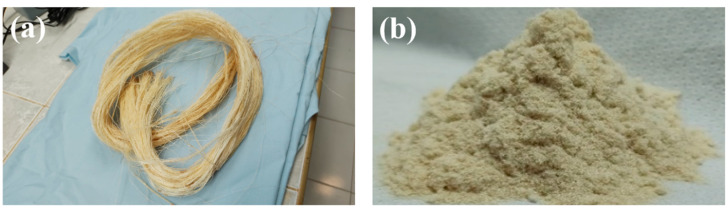
(**a**) henequen fibers; (**b**) henequen fiber flour.

Long henequen fibers (Figure 1a) were obtained from a producer in Baca, Yucatan (Desfibradora La Lupita, Baca, Yucatan, Mexico). The chemical composition of the henequen fiber is cellulose (60 wt%), hemicellulose (28 wt%), lignin (8 wt%), and extractives (4 wt%) [42], while its crystalline fraction is 0.4 [43]. The fibers were subsequently chopped to a length of 10 mm. First, the henequen fibers were reduced by a Brabender mill type 880804 (Brabender, Duisburg, Germany) to a maximum particle size of 600 μm. Next, the particles were reduced by a mill model Wiley Mini Mill (Thomas Scientific, Swedesboro, USA) to a maximum size of 250 μm. The flour was then sieved using a four-sieve column (250 μm, 180 μm, 150 μm, 90 μm) to obtain flour with particle sizes between 90 and 250 μm. The henequen flour (Figure 1b) was subsequently used to fabricate the NFRC filaments. The selected particle size range was based on preliminary results showing that particles larger than 250 μm affected the fabrication process of the filaments and 3D printed samples by obstructing the extruder die and the printer nozzle. In addition, the smallest particles (<90 μm) were discarded to obtain a more homogenous particle size distribution.

### 2.2. NFRC Filament Preparation

**Figure 2:**
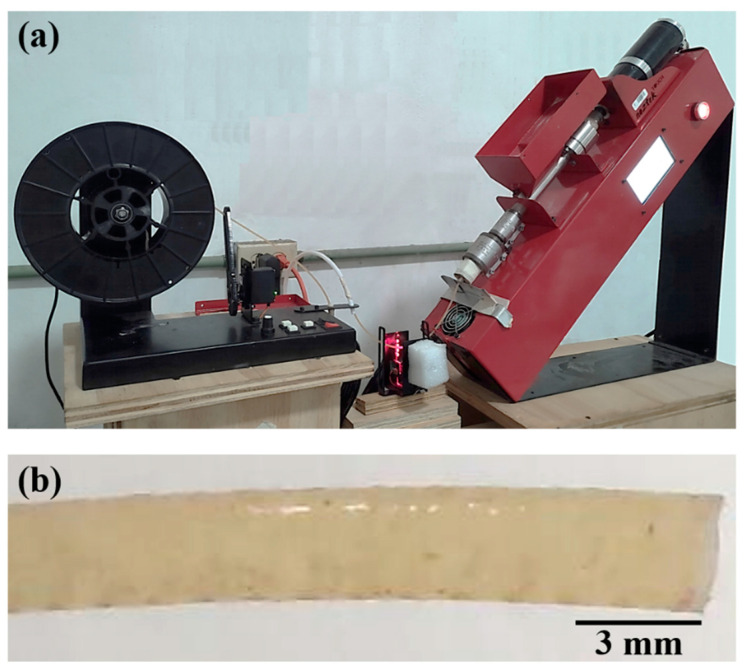
(**a**) Extrusion process of the NFRC filaments; (**b**) PLA/henequen filament with a fiber flour content of 3 wt%.

Before filament fabrication, PLA pellets were dried in an oven at 90 °C for 2 h, while the henequen flour was dried at 105 °C for 24 h. PLA/henequen NFRCs were prepared using a reinforcement content of 1–5 wt%. Table 1 shows the NFRC formulations. A single extrusion process [44] was used to fabricate the NFRC filaments. In this process, a mix of PLA pellets and flour was placed in the chamber of a single screw extruder Noztek Pro (Noztek, Shoreham-By-Sea, UK) equipped with a 2.8-mm diameter die, as shown in Figure 2a; subsequently, filaments were extruded using temperatures in the range of 175–185 °C at 15 rpm. The PLA/henequen extruded filaments (Figure 2b) had an average diameter of 2.78 ± 0.03 mm. As a reference, pristine PLA filaments were also fabricated following the abovementioned process without adding flour (Table 1). In addition, a formulation with 1 wt% content of fiber flour and 3 wt% content of maleic anhydride (Sigma–Aldrich Chem. Corp., St. Louis, MO, USA), employed as a coupling agent, was used to fabricate filaments (Table 1).

### 2.3. 3D Printing of NFRC Specimens

**Figure 3:**
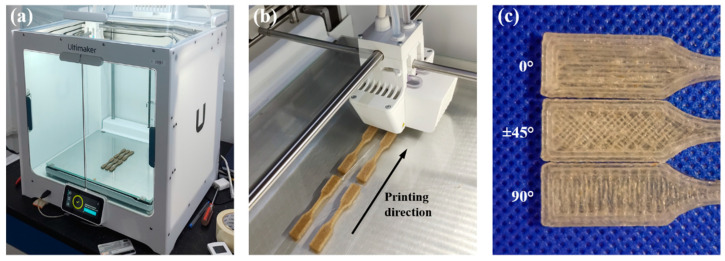
(**a**) 3D printer; (**b**) 3D printed NFRC tensile samples with a raster angle of 0°; (**c**) 3D printed NFRC tensile samples with raster angles of 0°, ±45° and 90° and with a fiber flour content of 1%.

NFRC specimens for the tensile tests were manufactured using the Ultimaker S5 3D printer (Ultimaker, Utrecht, The Netherlands) (Figure 3a) at a temperature of 215 °C, with a plate temperature of 60 °C, a filament feed speed of 30 mm/s, and an infill density of 100%. An infill pattern of “Lines” was selected in the Ultimaker Cura 4.11.0 software (Ultimaker, Utrecht, The Netherlands). A layer thickness of 0.15 mm was used to fabricate samples with a total thickness of 3.2 mm. The specimens were printed in a flat orientation (built direction) with a raster angle of 0° (Figure 3b); that is, the printing direction was parallel to the tensile testing direction (Figure 3b). In addition, a 0.8-mm diameter printing nozzle was used. The dimensions of the 3D printed samples were according to the ASTM D638 standard (Type V specimens), which had a gauge length of 12 mm and a cross-sectional area of 3.18 × 3.2 mm^2^ in the gauge region. In addition, specimens with a PLA/H1 formulation (Table 1) were printed with raster angles of ±45° and 90° (Figure 3c).

### 2.4. Scanning Electron Microscopy (SEM)

Morphological observations were carried out by scanning electron microscopy (SEM) using an SEM microscope Jeol JSM 6360LV (JEOL, Tokyo, Japan). All samples were coated with a thin layer of gold for electron conductivity. In addition, SEM observations were conducted on tested specimens to observe the fracture characteristics.

### 2.5. Density and Porosity

Printed pristine PLA and NFRCs densities were measured using a Techne density gradient column (Cole-Parmer, Vernon Hills, IL, USA) filled with an aqueous solution of calcium nitrate at 23 °C [45,46,47]. The porosity *P* of the printed PLA and NFRCs was calculated using the following equation:

(1) $$P\left(\%\right)=\left(1-\frac{\rho_{m}}{\rho_{t}}\right)\times 100$$

where $\rho_{m}$ is the measured density of the printed PLA and NFRCs, while $\rho_{t}$ is the theoretical density of the printed PLA and NFRCs, which was estimated using the following equation:

(2) $$\rho_{t}=\frac{1}{\frac{W_{PLA}}{\rho_{PLA}}+\frac{W_{H}}{\rho_{H}}}$$

where $\rho_{PLA}$ is the solid density of the PLA reported by the manufacturer (1240 kg/m^3^), $\rho_{H}$ is the density of the henequen fiber reported as 1570 kg/m^3^ [43], and $W_{PLA}$ and $W_{H}$ are the weight fractions of PLA and henequen-fiber flour, respectively.

### 2.6. Differential Scanning Calorimetry (DSC) Analysis

A differential scanning calorimetry (DSC) analysis of printed PLA and NFRCs was performed using a DSC 7 calorimeter (Perkin-Elmer, Waltham, MA, USA) to study NFRCs’ melting behavior and crystallinity. Only the first heating was performed from room temperature to 220 °C at a heating rate of 5 °C/min under an inert nitrogen atmosphere. The cold crystallization temperature $T_{cc}$ and melting temperature $T_{m}$ were recorded during the first heating. The degree of crystallinity $X_{c}$ was calculated during the first heating to determine the effect of the printing process on the crystallinity of the printed PLA and NFRCs. A similar approach has been used previously, in which only the first heating curves were recorded to correlate the degree of crystallinity resulting from the 3D printing process with the mechanical properties without eliminating the thermal history [48,49]. $X_{c}$ was calculated using the following equation:

(3) $$X_{c}=\frac{\Delta H_{m}-\Delta H_{cc}}{\Delta H_{mo}\times W_{PLA}}$$

where $\Delta H_{m}$ is the melting enthalpy, $\Delta H_{cc}$ is the cold crystallization enthalpy, $\Delta H_{mo}=93\;J/g$ is the melting enthalpy of 100% crystalline PLA [50], and *W_PLA_* is the weight fraction of PLA in the sample.

### 2.7. Tensile Testing

Uniaxial tensile tests were performed using a universal testing machine Shimadzu AGX-10 (Shimadzu, Kyoto, Japan), equipped with a 1 kN load cell at a crosshead speed of 1 mm/min at room temperature. The tensile tests were carried out according to the ASTM D638 standard.

### 2.8. Statistical Analysis

The results are presented as the mean ± standard deviation of at least three repetitions. Statistical analysis was carried out by a one-way analysis of variance (ANOVA) followed by Tukey’s multiple comparison test, which was performed using the stats.tukey_hsd function from the SciPy Python package [51]. It was considered a significant difference between means when *P* ≤ 0.05. For the DSC analysis, only one sample of each type of printed material was tested; thus, no statistical analysis of the DSC results is presented.

## 3. Results and Discussion

### 3.1. Particle Size Distribution

**Figure 4:**
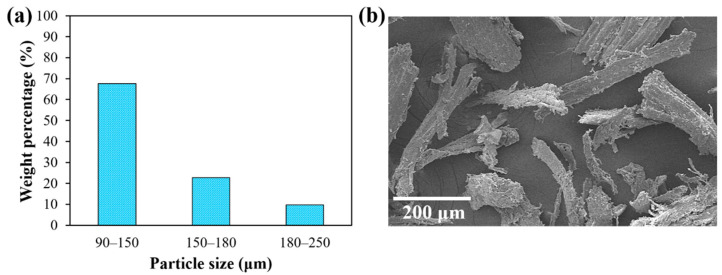
(**a**) Weight percentage of the particle sizes of the henequen flour; (**b**) SEM image of the henequen flour particles.

Figure 4a shows the weight percentage of the particle sizes of the henequen flour used to fabricate the NFRCs. The particle sizes were between 90 and 250 μm, as aforementioned. It can be seen in Figure 4a that the distribution is skewed left, and most particle sizes were between 90 and 150 µm. The henequen flour comprised a more significant percentage (67%) of small particles (90–150 μm). Conversely, the percentage of large particles (180–250 μm) was only 10%. The estimated mean particle size from Figure 4a is 140 μm. Figure 4b shows an SEM image of the henequen flour particles; it can be seen that the particles are mainly flat and elongated in shape.

### 3.2. Density and Porosity

**Figure 5:**
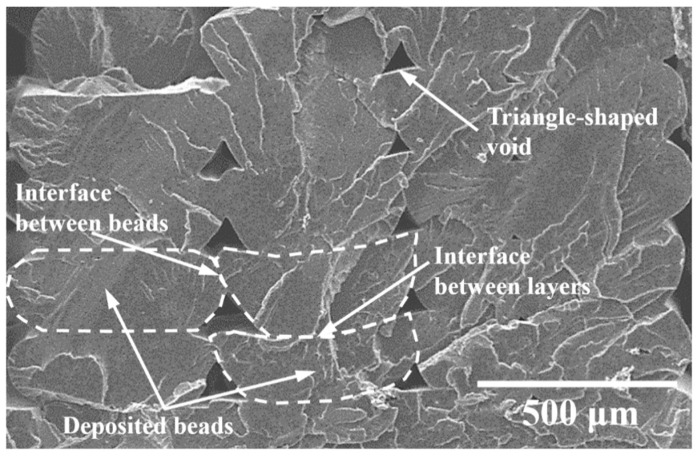
SEM image of the voids formed during the printing process of PLA.

Table 2 shows the theoretical and measured densities and calculated porosity (using Equation (1)) for the printed PLA and NFRCs. As expected, the density increases with the increase in henequen-flour content, considering that the henequen fiber has a larger density (1570 kg/m^3^) than the PLA (1240 kg/m^3^). However, it can also be seen that the measured density is lower than the theoretical density, which is also expected considering the voids between adjacent layers and deposited beads generated during the printing process (Figure 5), which largely contributed to the porosity of the printed materials. The SEM image in Figure 5 was taken from the surface of a printed PLA sample fractured in liquid nitrogen. The shape of the voids observed in Figure 5 resemble triangles, which shows that the voids were formed during the printing process [52]; the voids’ size depends on the printing conditions, such as printing speed and nozzle or plate temperature [53]. These triangle-shaped voids have also been observed in 3D printed PLA/glass fiber composites [17]. The porosity of the printed PLA due to the triangle-shaped voids between layers and beads was roughly estimated as 4–5% by measuring the area of the voids in the SEM images (Figure 5) using the image-processing software ImageJ v. 1.60 (National Institutes of Health, Bethesda, MD, USA). The estimated porosity of the pristine PLA using the SEM images (4–5%) was lower than the calculated porosity (Table 2). This observation suggests that there are voids and defects not visible in the SEM images, which could have formed during the printing process due to the non-uniform surface of the NFRC filaments produced by the henequen particles. This rough surface could have contributed to the formation of air gaps between layers or adjacent deposited beads [54].

Furthermore, it is noted that the calculated porosity in Table 2 tends to increase with the increase in henequen flour content slightly. This observation could be explained by the fact that a chemical incompatibility exists between the hydrophobic molecules of thermoplastics and the hydrophilic lignocellulosic molecules of natural fibers [55,56], which could likely produce voids between the PLA matrix and the henequen fibers. In addition, for a higher content of henequen flour, fiber agglomeration is also expected, which could contribute to an increase in the porosity of the NFRCs. 

### 3.3. DSC Analysis

**Figure 6:**
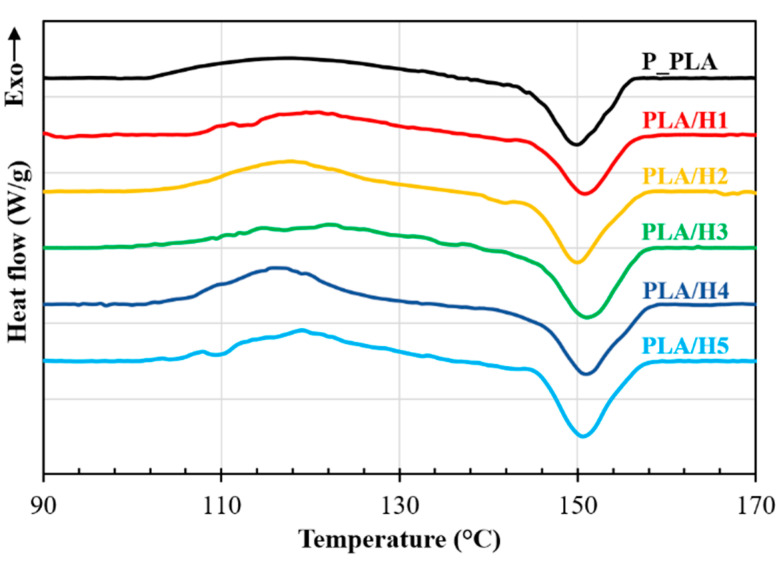
DSC heating curves of 3D printed PLA and NFRC specimens.

Figure 6 shows the DSC heating curves of the printed PLA and NFRC specimens, while the corresponding thermal parameters are summarized in Table 3. The first peak at ~120 °C (Figure 6) corresponds to a broad exothermic transition of low intensity associated with the cold crystallization of a semicrystalline polymer [57]. In addition, this peak shows that during PLA and NFRC heating, a reorganization of the amorphous domains could occur, promoting crystalline structure formation. The second peak, at higher temperatures (~150 °C), corresponds to an exothermic transition indicating the melting of the crystalline structures produced during the cooling of 3D printed samples and the cold crystallization. 

It can be seen in Table 3 that the degree of crystallinity of the printed pristine PLA is 1.45%, which is similar to the reported value for printed PLA 2003D [48]. This low degree of crystallinity is attributed to the high levels of D-isomer content [58]. It can also be seen in Table 3 that the degree of crystallinity slightly increases with the increase in henequen flour content. This increase could be attributed to the presence of the lignocellulosic material in the NFRCs. The natural henequen fiber can act as a nucleation agent facilitating the crystallization of the PLA [59]. 

### 3.4. Tensile Tests

**Figure 7:**
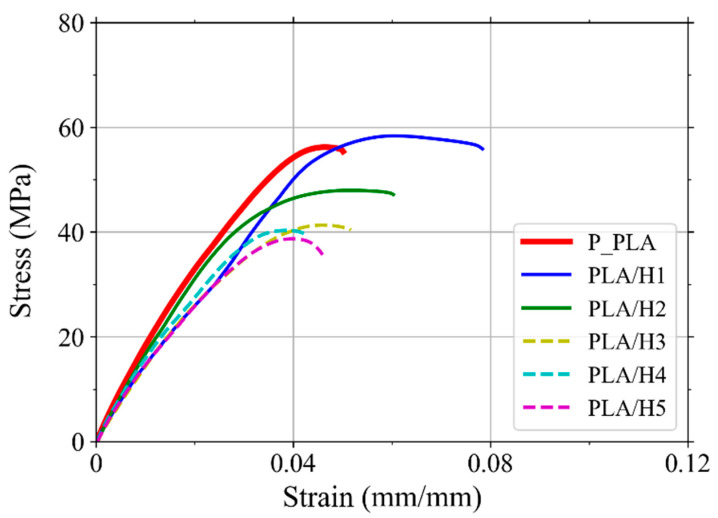
Average tensile stress–strain curves of 3D printed PLA and NFRCs with different contents of henequen flour.

Figure 7 shows the average tensile stress–strain curves of pristine PLA and NFRC specimens printed with a raster angle of 0°. It can be seen that most of the samples exhibited an initial linear elastic behavior up to around 40 MPa, followed by a non-linear region until the maximum stress was reached. After the maximum stress peak, a reduction in stress with increasing strain was observed until failure occurred. 

Table 4 shows the tensile Young’s modulus, maximum stress, and strain to failure of the printed PLA and NFRCs obtained from the stress–strain curves. It is noted that the Young’s modulus of the printed pristine PLA is 1.93 GPa, which is much lower than the value reported by the manufacturer (3.5 GPa). This reduction in mechanical properties could be explained by the void formation between printed layers and beads, increased porosity in the material produced by the printing process [60], and the reduction in the molecular weight due to the extrusion and printing processes [61,62]. In addition, debonding between adjacent deposited beads (Figure 5) [63] and changes in the degree of crystallinity during the FDM process [24] could also have affected the material’s mechanical performance. It can be seen in Table 4 that the Young’s modulus of the NFRCs is lower than that of the printed pristine PLA. This observation is explained by the fact that adding flour to the PLA leads to particle agglomeration and voids, reducing the material’s stiffness [64]. 

**Figure 8:**
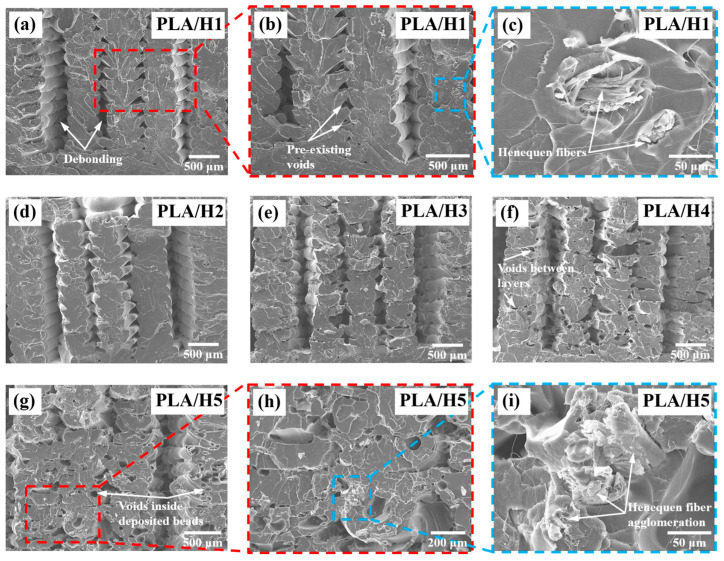
SEM images of the fracture surfaces after tensile testing of the 3D printed NFRCs: (**a**) PLA/H1; (**b**) PLA/H1 (close-up view); (**c**) PLA/H1 (close-up view); (**d**) PLA/H2; (**e**) PLA/H3; (**f**) PLA/H4; (**g**) PLA/H5; (**h**) PLA/H5 (close-up view); (**i**) PLA/H5 (close-up view).

It can be seen in Table 4 that the maximum stress of the printed PLA (56.7 MPa) is slightly lower than the value reported by the manufacturer (60 MPa), which is attributed to the triangle-shaped voids between layers [65]. However, for PLA/H1, the maximum stress is 60 MPa, which is 5.3% higher than that of the printed pristine PLA. Moreover, the strain to failure of the PLA/H1 is 0.078 (Figure 8c), which is 53% higher than that of the printed pristine PLA (0.051). This improvement in strength and ductility is attributed to the henequen fibers acting as reinforcement and delaying crack growth. Furthermore, for flour contents of 2% and higher, the maximum stress of the NFRCs is lower than those of the printed pristine PLA and PLA/H1. Moreover, the strain to failure decreases with the increase in flour content. This observation is attributed to fiber agglomeration and increased porosity.

Figure 8 shows SEM images of the fracture surfaces after the tensile testing of the 3D printed NFRCs. It can be seen that in all cases, there is debonding between adjacent beads (Figure 8a), which is attributed to insufficient bonding produced during the FDM process and stress concentration generated at pre-existing voids formed during the FDM process [53]. This type of debonding has been previously reported for printed PLA [66]. The debonding may have been caused by the presence of voids and bead shrinkage induced by the semicrystalline nature of the PLA [49], resulting in insufficient surface contact between adjacent beads [67]. This, in turn, may have produced a weak bond between adjacent beads leading to debonding when the load was applied to the specimens. In addition, it can be seen in Figure 8 that there is an increase in the apparent surface roughness with the increase in flour content attributed to the henequen particles (Figure 8c). This observation is more evident for flour contents of 4% and 5%, for which the surface is rough and has several voids (Figure 8f,g). These voids include those formed between deposited beads during the FDM process and those between layers (Figure 8f). The latter void formation is attributed to large particles and particle agglomeration. Finally, voids are also observed inside the deposited beads, attributed again to the large particles (Figure 8g) and fiber agglomeration (Figure 8i). This increase in defects and fiber agglomeration with the increase in flour content will ultimately affect the mechanical properties of the NFRCs. 

#### Effect of Printing Direction and Coupling Agent

**Figure 9:**
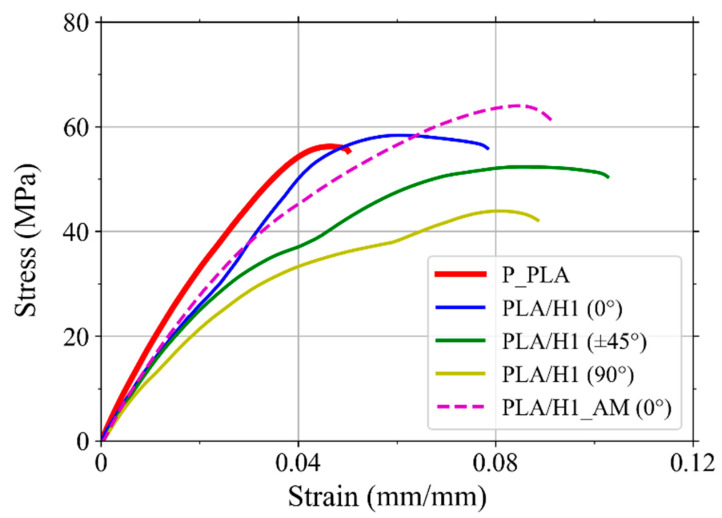
Average tensile stress–strain curves of 3D printed P_PLA and PLA/H1_MA specimens, and PLA/H1 specimens printed with raster angles of 0°, ±45°, and 90°.

Figure 9 shows the average tensile stress–strain curves of the pristine PLA specimens, PLA/H1_MA specimens, and PLA/H1 specimens printed with raster angles of 0°, ±45°, and 90°, while Table 5 shows the mechanical properties obtained from the stress-strain curves. It can be seen in Figure 9 that for the specimens printed with a raster angle of ±45°, there was a reduction in Young’s modulus and maximum stress compared to the specimens printed with a raster angle of 0°. Furthermore, the specimens with a raster angle of ±90° exhibited a further reduction in Young’s modulus and maximum stress compared to the other two printing orientations. However, the samples printed with raster angles of ±45° and 90° exhibited higher strain to failure than the specimens printed with a raster angle of 0°. These observations are in agreement with the results obtained for PLA printed with raster angles of 0°, ±45°, and 90°, in which the specimens printed with a raster angle of 0° exhibited the best mechanical performance, while the samples printed with a raster angle of 90° showed the worse mechanical performance [68,69]. This observation is explained by the fact that the individual beads of the specimens printed with a raster angle of 90° are perpendicular to the tensile load; thus, the fracture of the specimen is mainly determined by the bonding between adjacent beads [68].

It can be seen in Figure 9 that the specimens with a coupling agent (PLA/H1_AM) exhibited a similar Young’s modulus to that of the samples without a coupling agent (PLA/H1) and lower than that of the pristine PLA (P_PLA). It can also be seen that the PLA/H1_AM specimens exhibited higher maximum stress and strain to failure than the P_PLA and PLA/H1 specimens; however, further research should be performed to confirm these improved properties.

### 3.5. Discussion

The results presented here have shown that henequen fibers can be used to fabricate 3D printed PLA-based composites with improved mechanical properties using natural resources. These composites, made from renewable materials, are environmentally friendly and could promote sustainability. In addition, the results showed that printed PLA reinforced with 1% henequen flour exhibited higher maximum stress and ductility than printed PLA without reinforcement.

However, one of the main concerns in developing these printed NFRCs is the chemical incompatibility between the hydrophilic lignocellulosic henequen fiber and the hydrophobic PLA matrix, which produces poor adhesion [70]. The results showed a decrease in mechanical properties for flour contents of 2% and higher, which was partly attributed to the poor adhesion between the PLA and the henequen fibers; this poor adhesion could, in turn, have produced fiber agglomeration [71]. Therefore, a strong adhesion at the fiber/matrix interface is desirable for the effective transfer of stresses from the matrix to the reinforcement, resulting in better mechanical properties for the composite. For this reason, using methods to promote henequen particles’ interfacial bonding is essential to improve the performance of these NFRCs. Using a chemical alkaline treatment on the henequen fiber followed by surface treatment with a silane coupling agent could enhance the interface adhesion [40,72]. Thermal treatments could also improve the adhesion between the henequen fiber and the PLA matrix by removing hydrophilic materials such as hemicellulose [73]. Finally, a coupling agent, such as maleic anhydride, could be used to improve the PLA matrix–henequen fiber adhesion and, thus, the mechanical properties of the NFRCs [74]. 

The NFRCs mechanical results, along with the SEM images of fracture surfaces, suggest that with the increase in henequen-flour content (>2%), there is an increase in porosity and void formation, which may be attributed to particle agglomeration resulting in a reduction in mechanical properties. However, further studies should be performed to confirm these observations and to understand how flour content affects the overall performance of the NRCFs. A careful selection of particle sizes and a double extrusion process [44] could increase the NFRC filaments’ homogeneity and particle dispersion. These enhancements could, in turn, improve their printability and allow higher flour content incorporation without compromising mechanical performance. 

The SEM images showed that the NFRCs exhibited debonding between adjacent deposited beads, mainly attributed to interfacial defects and voids formed during the printing process; these defects ultimately acted as stress concentrators and points of failure initiation. The presence of voids and bead shrinkage caused by the semicrystalline nature of the PLA [67] may have resulted in insufficient surface contact between beads and poor bonding, resulting in a reduction in mechanical properties due to debonding when the load was applied. It has been reported that increasing the contact area between adjacent beads and using slow cooling rates [75] can reduce thermal gradient and promote molecular diffusion [76], resulting in improved bonding and increased mechanical properties. In addition, using an increased build plate temperature can minimize the adverse effects of thermal gradients on bonding [49]. 

It has been reported that the infill pattern used during the 3D printing of PLA specimens affects their mechanical properties [77]. For instance, it was reported that PLA tensile specimens printed with a flat orientation showed the highest maximum tensile strength when linear and concentric infill patterns were used compared to a Hilbert curve infill pattern [78]. Moreover, it was found that the concentric infill pattern produced the highest tensile properties in 3D printed PLA specimens compared to the grid and tri-hexagonal patterns [79]. For these reasons, the infill pattern should be considered an important factor when designing 3D printed parts to obtain optimal mechanical properties. 

Therefore, it is recommended to investigate different parameters that could improve the printing quality and influence the printed NFRCs’ mechanical performance, such as processing temperatures, layer orientation, deposition speed, infill pattern, and layer thickness [80]. Furthermore, our results warrant further research of NFRCs made of printed PLA reinforced with natural henequen fibers for construction and automotive applications [16,81]. These studies should include strategies to improve fiber–matrix adhesion and reduce fiber agglomeration during the filament extrusion process and defects during the printing process. In addition, further mechanical testing, including bending and compression tests, should be performed to fully understand the mechanical behavior of NFRCs.

## 4. Conclusions

In this work, natural fiber-reinforced composite (NFRC) filaments for 3D printing were fabricated using PLA reinforced with 1–5 wt% flour from henequen fibers. The filaments were subsequently used for the 3D printing of specimens for tension tests with a 0° raster angle. The effect of the flour content on the tensile properties was evaluated. Thermal, physical, and microscopic characterizations of the printed materials were also performed. In addition, for 1 wt% flour content, the NFRCs were also printed with raster angles of ±45° and 90°. Finally, an NFRC formulation with 3 wt% content of maleic anhydride and 1 wt% flour content was investigated. The following conclusions can be drawn from the results of this study:

- The henequen fiber flour used to fabricate the NFRCs consisted of particles with sizes between 90–250 μm comprising a more significant percentage (67%) of small particles (90–150 μm).
- The measured density and porosity of the NFRCs increased with the increase in flour content.
- Thermal analysis by DSC showed that adding henequen flour to the PLA matrix increased the NFRCs’ degree of crystallinity.
- The Young’s modulus of the NFRCs was lower than that of the printed pristine PLA, which was attributed to particle agglomeration and voids.
- The maximum stress and strain to failure of the NFRC with 1% flour content were higher than those of the printed PLA, attributed to the henequen fibers acting as reinforcement and delaying crack growth.
- However, for 2% and higher flour contents, the NFRCs’ maximum stress was lower than those of the printed pristine PLA and PLA with 1% flour content.
- Microscopic characterization of NFRCs after tensile testing showed an increase in voids and defects with the increase in flour content, which was attributed to particle agglomeration in the material.
- For 1 wt% flour content, the highest tensile properties were obtained with a 0° raster angle.
- Adding 3 wt% content of maleic anhydride to the NFRC with 1 wt% flour content slightly increased the maximum stress.
- The mechanical results indicated that printed NFRCs made of PLA reinforced with henequen fiber have the potential to be used as sustainable materials with improved mechanical properties; however, further research is still required to understand the mechanical performance of these materials fully.

## Figures and Tables

**Table 1:** Formulation of the filaments for 3D printing of PLA and NFRCs.

Material	Flour Content (wt%)	PLA Content (wt%)	Nomenclature
Pristine PLA	0	100	P_PLA
PLA/Henequen	1	99	PLA/H1
	2	98	PLA/H2
	3	97	PLA/H3
	4	96	PLA/H4
	5	95	PLA/H5
PLA/Henequen/maleic anhydride ^1^	1	96	PLA/H1_MA

^1^ With a maleic anhydride content of 3 wt%.

**Table 2:** Density and porosity values of the 3D printed materials.

Material	Theoretical Density (kg/m^3^)	Measured Density (kg/m^3^)	Porosity *P* (%)
P_PLA	1240.0	1154.4 ± 0.5 ^d^	6.984 ± 0.047 ^c^
PLA/H1	1242.6	1158.8 ± 0.3 ^c^	6.825 ± 0.028 ^d^
PLA/H2	1245.2	1162.3 ± 0.5 ^b^	6.740 ± 0.046 ^d^
PLA/H3	1247.9	1163.1 ± 0.4 ^b^	6.873 ± 0.036 ^c^
PLA/H4	1250.5	1159.6 ± 0.4 ^c^	7.350 ± 0.037 ^a^
PLA/H5	1253.2	1165.8 ± 0.5 ^a^	7.052 ± 0.046 ^b^

Note: Values in a column with different superscript letters are significantly different (P < 0.05).

**Table 3:** Thermal properties and degree of crystallinity of 3D printed PLA and NFRCs.

Material	Cold Crystallization Temperature $T_{cc}\;({}^{\circ}C)$	Cold Crystallization Enthalpy $\Delta H_{cc}\;(J/g)$	Melting Temperature $T_{m}\;(\text{°}C)$	Melting Enthalpy $\Delta H_{m}\;(J/g)$	Degree of Crystallinity $X_{c}\;(\%)$
P_PLA	117.8	11.523	150.1	12.881	1.45
PLA/H1	119.9	9.781	150.9	11.184	1.59
PLA/H2	118.0	12.744	150.0	14.293	1.65
PLA/H3	122.4	12.445	150.9	14.271	1.95
PLA/H4	116.7	13.384	151.2	15.153	1.89
PLA/H5	119.0	12.487	150.5	14.437	2.08

**Table 4:** Tensile mechanical properties of 3D printed PLA and NFRCs.

Material	Young’s Modulus (GPa)	Maximum Stress (MPa)	Strain to Failure (mm/mm)
P_PLA	1.93 ± 0.10 ^a^	56.7 ± 2.3 ^a^	0.051 ± 0.008 ^b^
PLA/H1	1.48 ± 0.19 ^b^	60.1 ± 1.8 ^a^	0.078 ± 0.010 ^a^
PLA/H2	1.62 ± 0.11 ^ab^	48.9 ± 0.9 ^b^	0.060 ± 0.018 ^ab^
PLA/H3	1.43 ± 0.06 ^b^	41.4 ± 1.1 ^c^	0.052 ± 0.003 ^b^
PLA/H4	1.58 ± 0.07 ^b^	40.4 ± 1.2 ^c^	0.043 ± 0.002 ^b^
PLA/H5	1.49 ± 0.11 ^b^	35.5 ± 5.3 ^c^	0.045 ± 0.004 ^b^

Note: Values in a column with different superscript letters are significantly different (P < 0.05).

**Table 5:** Tensile mechanical properties of 3D printed P_PLA and PLA/H1_MA specimens, and PLA/H1 specimens printed with raster angles of 0°, ±45°, and 90°.

Material	Young’s Modulus (GPa)	Maximum Stress (MPa)	Strain to Failure (mm/mm)
P_PLA	1.93 ± 0.10 ^a^	56.7 ± 2.3 ^ab^	0.051 ± 0.008 ^b^
PLA/H1 (0°)	1.48 ± 0.19 ^b^	60.1 ± 1.8 ^a^	0.078 ± 0.010 ^ab^
PLA/H1 (±45°)	1.37 ± 0.06 ^b^	51.2 ± 4.4 ^bc^	0.109 ± 0.019 ^a^
PLA/H1 (90°)	1.25 ± 0.11 ^b^	43.9 ± 2.4 ^c^	0.089 ± 0.021 ^a^
PLA/H1_MA	1.48 ± 0.07 ^b^	63.9 ± 3.7 ^a^	0.091 ± 0.006 ^a^

Note: Values in a column with different superscript letters are significantly different (P < 0.05).

## Data Availability

The data presented in this study are available on request from the corresponding author.

## References

[1] Ngo T.D., Kashani A., Imbalzano G., Nguyen K.T.Q., Hui D. (2018). Additive manufacturing (3D printing): A review of materials, methods, applications and challenges. Compos. Part B.

[2] Dizon J.R.C., Espera A.H., Chen Q., Advincula R.C. (2018). Mechanical characterization of 3D-printed polymers. Addit. Manuf..

[3] Mustapha K.B., Metwalli K.M. (2021). A review of fused deposition modelling for 3D printing of smart polymeric materials and composites. Eur. Polym. J..

[4] Tan L.J., Zhu W., Zhou K. (2020). Recent Progress on Polymer Materials for Additive Manufacturing. Adv. Funct. Mater..

[5] Bhagia S., Bornani K., Agrawal R., Satlewal A., Ďurkovič J., Lagaňa R., Bhagia M., Yoo C.G., Zhao X., Kunc V. (2021). Critical review of FDM 3D printing of PLA biocomposites filled with biomass resources, characterization, biodegradability, upcycling and opportunities for biorefineries. Appl. Mater. Today.

[6] Shahrubudin N., Lee T.C., Ramlan R. (2019). An Overview on 3D Printing Technology: Technological, Materials, and Applications. Procedia Manuf..

[7] Arefin A.M.E., Khatri N.R., Kulkarni N., Egan P.F. (2021). Polymer 3D Printing Review: Materials, Process, and Design Strategies for Medical Applications. Polymers.

[8] Djukić-Vuković A., Mladenović D., Ivanović J., Pejin J., Mojović L. (2019). Towards sustainability of lactic acid and poly-lactic acid polymers production. Renew. Sustain. Energy Rev..

[9] Balla E., Daniilidis V., Karlioti G., Kalamas T., Stefanidou M., Bikiaris N.D., Vlachopoulos A., Koumentakou I., Bikiaris D.N. (2021). Poly(lactic Acid): A Versatile Biobased Polymer for the Future with Multifunctional Properties—From Monomer Synthesis, Polymerization Techniques and Molecular Weight Increase to PLA Applications. Polymers.

[10] Ruz-Cruz M.A., Herrera-Franco P.J., Flores-Johnson E.A., Moreno-Chulim M.V., Galera-Manzano L.M., Valadez-González A. (2022). Thermal and mechanical properties of PLA-based multiscale cellulosic biocomposites. J. Mater. Res. Technol..

[11] Ayrilmis N. (2018). Effect of layer thickness on surface properties of 3D printed materials produced from wood flour/PLA filament. Polym. Test..

[12] Girdis J., Gaudion L., Proust G., Löschke S., Dong A. (2017). Rethinking Timber: Investigation into the Use of Waste Macadamia Nut Shells for Additive Manufacturing. JOM.

[13] Le Duigou A., Correa D., Ueda M., Matsuzaki R., Castro M. (2020). A review of 3D and 4D printing of natural fibre biocomposites. Mater. Des..

[14] Rajendran Royan N.R., Leong J.S., Chan W.N., Tan J.R., Shamsuddin Z.S. (2021). Current State and Challenges of Natural Fibre-Reinforced Polymer Composites as Feeder in FDM-Based 3D Printing. Polymers.

[15] Deb D., Jafferson J.M. (2021). Natural fibers reinforced FDM 3D printing filaments. Mater. Today. Proc..

[16] Khalid M.Y., Al Rashid A., Arif Z.U., Ahmed W., Arshad H., Zaidi A.A. (2021). Natural fiber reinforced composites: Sustainable materials for emerging applications. Results Eng..

[17] Nicolau A., Pop M.A., Coșereanu C. (2022). 3D Printing Application in Wood Furniture Components Assembling. Materials.

[18] Csizmadia R., Faludi G., Renner K., Móczó J., Pukánszky B. (2013). PLA/wood biocomposites: Improving composite strength by chemical treatment of the fibers. Compos. Part A.

[19] Kazemi Najafi S. (2013). Use of recycled plastics in wood plastic composites—A review. Waste Manag..

[20] Balla V.K., Kate K.H., Satyavolu J., Singh P., Tadimeti J.G.D. (2019). Additive manufacturing of natural fiber reinforced polymer composites: Processing and prospects. Compos. Part B.

[21] Mazzanti V., Mollica F. (2020). A Review of Wood Polymer Composites Rheology and Its Implications for Processing. Polymers.

[22] Elsheikh A.H., Panchal H., Shanmugan S., Muthuramalingam T., El-Kassas A.M., Ramesh B. (2022). Recent progresses in wood-plastic composites: Pre-processing treatments, manufacturing techniques, recyclability and eco-friendly assessment. Clean. Eng. Technol..

[23] Lee C.H., Padzil F.N.B.M., Lee S.H., Ainun Z.M.A., Abdullah L.C. (2021). Potential for Natural Fiber Reinforcement in PLA Polymer Filaments for Fused Deposition Modeling (FDM) Additive Manufacturing: A Review. Polymers.

[24] Figueroa-Velarde V., Diaz-Vidal T., Cisneros-López E.O., Robledo-Ortiz J.R., López-Naranjo E.J., Ortega-Gudiño P., Rosales-Rivera L.C. (2021). Mechanical and Physicochemical Properties of 3D-Printed Agave Fibers/Poly(lactic) Acid Biocomposites. Materials.

[25] Matsuzaki R., Ueda M., Namiki M., Jeong T.-K., Asahara H., Horiguchi K., Nakamura T., Todoroki A., Hirano Y. (2016). Three-dimensional printing of continuous-fiber composites by in-nozzle impregnation. Sci. Rep..

[26] Hinchcliffe S.A., Hess K.M., Srubar W.V. (2016). Experimental and theoretical investigation of prestressed natural fiber-reinforced polylactic acid (PLA) composite materials. Compos. Part B.

[27] Depuydt D., Balthazar M., Hendrickx K., Six W., Ferraris E., Desplentere F., Ivens J., Van Vuure A.W. (2019). Production and characterization of bamboo and flax fiber reinforced polylactic acid filaments for fused deposition modeling (FDM). Polym. Compos..

[28] Liu L., Lin M., Xu Z., Lin M. (2019). Polylactic Acid-based Wood-plastic 3D Printing Composite and its Properties. Bioresources.

[29] Bhagia S., Lowden R.R., Erdman D., Rodriguez M., Haga B.A., Solano I.R.M., Gallego N.C., Pu Y., Muchero W., Kunc V. (2020). Tensile properties of 3D-printed wood-filled PLA materials using poplar trees. Appl. Mater. Today.

[30] Kariz M., Sernek M., Obućina M., Kuzman M.K. (2018). Effect of wood content in FDM filament on properties of 3D printed parts. Mater. Today Commun..

[31] Kariz M., Sernek M., Kuzman M.K. (2018). Effect of humidity on 3D-printed specimens from wood-PLA filaments. Wood Res..

[32] Tao Y., Wang H., Li Z., Li P., Shi S.Q. (2017). Development and Application of Wood Flour-Filled Polylactic Acid Composite Filament for 3D Printing. Materials.

[33] Moscoso Sánchez F.J., Alvarado A., Martínez-Chávez L., Hernández-Montelongo R., Fernández Escamilla V.V., Canche Escamilla G. (2019). The effects of henequen cellulose treated with polyethylene glycol on properties of polylactic acid composites. Bioresources.

[34] Tarrés Q., Vilaseca F., Herrera-Franco P.J., Espinach F.X., Delgado-Aguilar M., Mutjé P. (2019). Interface and micromechanical characterization of tensile strength of bio-based composites from polypropylene and henequen strands. Ind. Crops Prod..

[35] Kim J., Cho D. (2022). Effects of Alkali-Treatment and Feeding Route of Henequen Fiber on the Heat Deflection Temperature, Mechanical, and Impact Properties of Novel Henequen Fiber/Polyamide 6 Composites. J. Compos. Sci.

[36] Castillo-Lara J.F., Flores-Johnson E.A., Valadez-Gonzalez A., Herrera-Franco P.J., Carrillo J.G., Gonzalez-Chi P.I., Agaliotis E., Li Q.M. (2021). Mechanical behaviour of composite sandwich panels with foamed concrete core reinforced with natural fibre in four-point bending. Thin-Walled Struct..

[37] Castillo-Lara J.F., Flores-Johnson E.A., Valadez-Gonzalez A., Herrera-Franco P.J., Carrillo J.G., Gonzalez-Chi P.I., Li Q.M. (2020). Mechanical Properties of Natural Fiber Reinforced Foamed Concrete. Materials.

[38] Agaliotis E.M., Morales-Arias J.P., Bernal C.R. Morphological, Mechanical and Thermal Characterization of Intralayer Hybrid Composites Based on Polylactic Acid (PLA) and Sisal Fiber. J. Nat. Fibers.

[39] Mofokeng J.P., Luyt A.S., Tábi T., Kovács J. (2011). Comparison of injection moulded, natural fibre-reinforced composites with PP and PLA as matrices. J. Thermoplast. Compos. Mater..

[40] Valadez-Gonzalez A., Cervantes-Uc J.M., Olayo R., Herrera-Franco P.J. (1999). Chemical modification of henequén fibers with an organosilane coupling agent. Compos. Part B.

[41] INGEO (2020). Ingeo™ Biopolymer 2003D Technical Data Sheet.

[42] Valadez-Gonzalez A., Cervantes-Uc J.M., Olayo R., Herrera-Franco P.J. (1999). Effect of fiber surface treatment on the fiber–matrix bond strength of natural fiber reinforced composites. Compos. Part B.

[43] Aguilar-Vega M., Cruz-Ramos C.A. (1995). Properties of henequen cellulosic fibers. J. Appl. Polym. Sci..

[44] Huang Y., Löschke S., Proust G. (2021). In the mix: The effect of wood composition on the 3D printability and mechanical performance of wood-plastic composites. Compos. Part C.

[45] González-Díaz M.O., Sulub-Sulub R., Herrera-Kao W., Vázquez-Torres H., Zolotukhin M.G., Aguilar-Vega M. (2018). Enhanced Gas Transport Performance of Polyamide Membranes by Postpolymerization Modification. Ind. Eng. Chem. Res..

[46] González-Díaz M.O., Pérez-Francisco J.M., Herrera-Kao W., González-Díaz A., Montes-Luna A., Aguilar-Vega M. (2017). Novel copolyaramides with bulky flexible groups for pure and mixed-gas separation. Sep. Purif. Technol..

[47] Cetina-Mancilla E., González-Díaz M.O., Sulub-Sulub R., Zolotukhin M.G., González-Díaz A., Herrera-Kao W., Ruiz-Treviño F.A., Aguilar-Vega M. (2022). Aging resistant, fluorinated aromatic polymers with ladderized, rigid kink-structured backbones for gas separations. J. Membr. Sci..

[48] Coppola B., Cappetti N., Di Maio L., Scarfato P., Incarnato L. (2018). 3D Printing of PLA/clay Nanocomposites: Influence of Printing Temperature on Printed Samples Properties. Materials.

[49] Peng X., Zhang M., Guo Z., Sang L., Hou W. (2020). Investigation of processing parameters on tensile performance for FDM-printed carbon fiber reinforced polyamide 6 composites. Compos. Commun..

[50] Baghaei B., Skrifvars M., Salehi M., Bashir T., Rissanen M., Nousiainen P. (2014). Novel aligned hemp fibre reinforcement for structural biocomposites: Porosity, water absorption, mechanical performances and viscoelastic behaviour. Compos. Part A.

[51] Virtanen P., Gommers R., Oliphant T.E., Haberland M., Reddy T., Cournapeau D., Burovski E., Peterson P., Weckesser W., Bright J. (2020). SciPy 1. 0: Fundamental algorithms for scientific computing in Python. Nat. Methods.

[52] Tronvoll S.A., Vedvik N.P., Elverum C.W., Welo T. (2019). A new method for assessing anisotropy in fused deposition modeled parts using computed tomography data. Int. J. Adv. Manuf. Technol..

[53] Tao Y., Kong F., Li Z., Zhang J., Zhao X., Yin Q., Xing D., Li P. (2021). A review on voids of 3D printed parts by fused filament fabrication. J. Mater. Res. Technol..

[54] Blok L.G., Longana M.L., Yu H., Woods B.K.S. (2018). An investigation into 3D printing of fibre reinforced thermoplastic composites. Addit. Manuf..

[55] Sepe R., Bollino F., Boccarusso L., Caputo F. (2018). Influence of chemical treatments on mechanical properties of hemp fiber reinforced composites. Compos. Part B.

[56] Hao M., Wu H., Qiu F., Wang X. (2018). Interface Bond Improvement of Sisal Fibre Reinforced Polylactide Composites with Added Epoxy Oligomer. Materials.

[57] Giani N., Mazzocchetti L., Benelli T., Picchioni F., Giorgini L. (2022). Towards sustainability in 3D printing of thermoplastic composites: Evaluation of recycled carbon fibers as reinforcing agent for FDM filament production and 3D printing. Compos. Part A.

[58] Backes E.H., Pires L.d.N., Costa L.C., Passador F.R., Pessan L.A. (2019). Analysis of the Degradation During Melt Processing of PLA/Biosilicate^®^ Composites. J. Compos. Sci..

[59] Ye C., Ma G., Fu W., Wu H. (2015). Effect of fiber treatment on thermal properties and crystallization of sisal fiber reinforced polylactide composites. J. Reinf. Plast. Compos..

[60] Liao Y., Liu C., Coppola B., Barra G., Di Maio L., Incarnato L., Lafdi K. (2019). Effect of Porosity and Crystallinity on 3D Printed PLA Properties. Polymers.

[61] Carrasco F., Pagès P., Gámez-Pérez J., Santana O.O., Maspoch M.L. (2010). Processing of poly(lactic acid): Characterization of chemical structure, thermal stability and mechanical properties. Polym. Degrad. Stab..

[62] Zhao P., Rao C., Gu F., Sharmin N., Fu J. (2018). Close-looped recycling of polylactic acid used in 3D printing: An experimental investigation and life cycle assessment. J. Clean. Prod..

[63] Dawoud M., Taha I., Ebeid S.J. (2016). Mechanical behaviour of ABS: An experimental study using FDM and injection moulding techniques. J. Manuf. Processes.

[64] Garzon-Hernandez S., Garcia-Gonzalez D., Jérusalem A., Arias A. (2020). Design of FDM 3D printed polymers: An experimental-modelling methodology for the prediction of mechanical properties. Mater. Des..

[65] Liu H., He H., Peng X., Huang B., Li J. (2019). Three-dimensional printing of poly(lactic acid) bio-based composites with sugarcane bagasse fiber: Effect of printing orientation on tensile performance. Polym. Adv. Technol..

[66] Bochnia J., Blasiak M., Kozior T. (2021). A Comparative Study of the Mechanical Properties of FDM 3D Prints Made of PLA and Carbon Fiber-Reinforced PLA for Thin-Walled Applications. Materials.

[67] Gao X., Qi S., Kuang X., Su Y., Li J., Wang D. (2021). Fused filament fabrication of polymer materials: A review of interlayer bond. Addit. Manuf..

[68] Zhang X., Chen L., Mulholland T., Osswald T.A. (2019). Effects of raster angle on the mechanical properties of PLA and Al/PLA composite part produced by fused deposition modeling. Polym. Adv. Technol..

[69] Khosravani M.R., Berto F., Ayatollahi M.R., Reinicke T. (2022). Characterization of 3D-printed PLA parts with different raster orientations and printing speeds. Sci. Rep..

[70] Orue A., Jauregi A., Peña-Rodriguez C., Labidi J., Eceiza A., Arbelaiz A. (2015). The effect of surface modifications on sisal fiber properties and sisal/poly (lactic acid) interface adhesion. Compos. Part B.

[71] Sun Z. (2018). Progress in the research and applications of natural fiber-reinforced polymer matrix composites. Sci. Eng. Compos. Mater..

[72] May-Pat A., Valadez-González A., Herrera-Franco P.J. (2013). Effect of fiber surface treatments on the essential work of fracture of HDPE-continuous henequen fiber-reinforced composites. Polym. Test..

[73] Huang C.-J., Li X.-L., Zhang Y.-Q., Feng Y.-H., Qu J.-P., He H.-Z., Shen H.-Z. (2013). Properties of heat-treated sisal fiber/polylactide composites. J. Thermoplast. Compos. Mater..

[74] Dzul-Cervantes M., Herrera-Franco P.J., Tábi T., Valadez-Gonzalez A. (2017). Using Factorial Design Methodology to Assess PLA-g-Ma and Henequen Microfibrillated Cellulose Content on the Mechanical Properties of Poly(lactic acid) Composites. Int. J. Polym. Sci..

[75] Abbott A.C., Tandon G.P., Bradford R.L., Koerner H., Baur J.W. (2018). Process-structure-property effects on ABS bond strength in fused filament fabrication. Addit. Manuf..

[76] Radzuan N.A.M., Sulong A.B., Verma A., Muhamad N. (2021). Layup sequence and interfacial bonding of additively manufactured polymeric composite: A brief review. Nanotechnol. Rev..

[77] Sriya Ambati S., Ambatipudi R. (2022). Effect of infill density and infill pattern on the mechanical properties of 3D printed PLA parts. Mater. Today. Proc..

[78] Dave H.K., Patadiya N.H., Prajapati A.R., Rajpurohit S.R. (2019). Effect of infill pattern and infill density at varying part orientation on tensile properties of fused deposition modeling-printed poly-lactic acid part. Proc. Inst. Mech. Eng. Part C J. Mech. Eng. Sci..

[79] Rismalia M., Hidajat S.C., Permana I.G.R., Hadisujoto B., Muslimin M., Triawan F. (2019). Infill pattern and density effects on the tensile properties of 3D printed PLA material. J. Phys. Conf. Ser..

[80] Oviedo A.M., Puente A.H., Bernal C., Pérez E. (2020). Mechanical evaluation of polymeric filaments and their corresponding 3D printed samples. Polym. Test..

[81] Huda M.K., Widiastuti I. (2021). Natural Fiber Reinforced Polymer in Automotive Application: A Systematic Literature Review. J. Phys. Conf. Ser..

